# Assessing the Presence of Eco-Anxiety in the General Population: A Systematic Review, Meta-Analysis and Meta-Regression

**DOI:** 10.3390/healthcare13212716

**Published:** 2025-10-27

**Authors:** Francesca Gallè, Federica Valeriani, Andrea De Giorgi, Fabiano Grassi, Elisa Mazzeo, Christian Napoli, Carmela Protano

**Affiliations:** 1 Department of Medical, Movement and Wellbeing Sciences, University of Naples Parthenope, 80133 Naples, Italy; 2 Department of Movement, Human, and Health Sciences, University of Rome Foro Italico, 00135 Rome, Italy; federica.valeriani@uniroma4.it; 3 Department of Public Health and Infectious Diseases, Sapienza University of Rome, 00185 Rome, Italy; andrea.degiorgi@uniroma1.it (A.D.G.); fabiano.grassi@uniroma1.it (F.G.); elisa.mazzeo@uniroma1.it (E.M.); 4 Department of Medical Surgical Sciences and Translational Medicine, Sapienza University of Rome, 00189 Rome, Italy; christian.napoli@uniroma1.it; 5 National Institute for Health, Migration and Poverty (NIHMP), 00153 Rome, Italy

**Keywords:** anxiety, eco-anxiety, environment, climate change

## Abstract

**Background/Objectives**: Eco-anxiety is emerging as a response to worsening environmental conditions. However, several gaps hinder the estimation of this phenomenon worldwide. This review aims to provide a measure of eco-anxiety control by those factors that may affect its prevalence assessment. **Methods**: The review was conducted in accordance with the PRISMA guidelines, and the protocol was registered on PROSPERO (CRD42024556132). PubMed, Scopus, Web of Science, and PsycINFO databases were interrogated. Cross-sectional studies in English and Italian languages assessing eco-anxiety through validated questionnaires were considered. The quality assessment was conducted using the adapted Newcastle–Ottawa Quality Assessment Scale. **Results**: Sixty-nine articles published between 2020 and 2025 were included. Of these, 60 studies were meta-analyzed, for a total sample size exceeding 65,000 participants across different countries and cultural contexts. The overall pooled mean eco-anxiety level was approximately 34.8/100 (95% CI: 29.6–39.9), corresponding to a moderate level of eco-anxiety, with women scoring higher than men (*p* < 0.05). Assessment tool and country were also shown as significant predictors of eco-anxiety, while age did not seem to play a significant role. **Conclusions**: Though further rigorous research is needed in this field, focusing on these variables could help to design targeted strategies that address environmental concerns and support mental well-being and resilience towards environmental challenges.

## 1. Introduction

In recent decades, the increased frequency of phenomena related with global warming, such as heatwaves, rising sea levels, hurricanes and floods, have raised the general consciousness about the environmental, social and health consequences of climate change. Nowadays, there is increasing awareness that climate change is one of the most significant issues for human and planetary health [1,2,3]. The World Health Organization (WHO) identifies climate change as the greatest threat to both physical and mental health [4]. In fact, besides physical disease and displacement, it can generate a sense of uncertainty and fear about the future, which may contribute to determining anxiety, stress, and other mental health issues [5].

In this scenario, a new psychological phenomenon, so-called “eco-anxiety”, is now emerging. Due to the complex and multifaceted nature of this phenomenon as a psychological response to the ongoing environmental crisis, there is no one universally accepted definition of eco-anxiety. Terms such as Climate Change Anxiety (CCA), climate-related worry, environmental distress, ecological grief, and ecological stress frequently appear in the literature, leading to a range of interpretations and definitions. For instance, eco-anxiety has been described as “*extreme worry about current and future harm to the environment caused by climate change*” [6] and as “*a chronic fear of environmental doom*” [7,8]. Additionally, it is characterized as “*heightened emotional, mental, or somatic distress in response to dangerous changes in the climate system*” [9,10]. Steffen et al. [11] defined eco-anxiety as a form of chronic fear or worry about the environmental catastrophe or “*the generalized sense that the ecological foundations of existence are in the process of collapse*” [12]. In 2011, Albrecht outlined this phenomenon by defining the “*psychoterratic*” syndromes as mental health impacts of negative emotions triggered by perceived environmental factors and climate change [13]. In general, eco-anxiety describes a complex emotional state characterized by worry, fear, helplessness, and sometimes even despair about the health of the planet and its long-term consequences for life; it can also include functional impairment and rumination [14]. It represents a new dimension of the interaction between the environment and the human psyche. With the growth in global climate crises, eco-anxiety has become the object of increasing attention within the scientific and psychological communities [15]. Preliminary findings have indicated that climate change may contribute to mental health issues, including functional impairment, symptoms of depression, anxiety, stress, and insomnia [16,17] and, in some cases, it could be a part of a broader syndrome, characterized by constant worries and intrusive thoughts [18]. On the other hand, some authors consider the anxiety related to climate change as a normal response and, for certain individuals, a motivation to environmentally sustainable behaviors [12]. In a world where climate change, natural disasters, and their consequences appear increasingly unavoidable, it is essential to understand how this threat affects the human mind and how the psychological effects of environmental crisis can be mitigated. Therefore, identifying eco-anxiety and its correlates in different populations can be useful to counteract them. Several scales have been developed over the years to assess eco-anxiety and its dimensions [16,19,20,21,22]. However, although their interrelation has been sometimes shown, the available scales differ in focus and domains. For example, some of these tools are aimed at assessing “eco-anxiety”, meant as feeling anxious about ecological problems, while others focus on “climate anxiety”, intended as feeling anxious about the climate crisis [21]. Furthermore, some studies in this field are aimed at assessing functional impairment or negative emotions such as worry towards ecological problems, which are related but do not coincide with eco-anxiety; some others explore eco-anxiety by using not validated tools or adapted questions nested in other questionnaires [10,14]. Other Authors have tried to describe the phenomenon of eco-anxiety, exploring the available literature, but they had to deal with these issues [10,14,21]. In this confusing context, the approach offered by a narrative synthesis, and even by a systematic review, cannot be sufficient from an epidemiological perspective.

Therefore, the present review aims to quantify the eco-anxiety worldwide by using a more inclusive search and a meta-analytic approach. We systematically analyze the available literature in this field, considering only those studies that assessed eco-anxiety through validated tools to shed light on the dimensions of eco-anxiety in the general population and on those factors that may affect its prevalence. Then, we proceeded to a meta-regression of the results by considering these factors as possible predictors.

## 2. Materials and Methods

### 2.1. Search Strategy

This systematic review was carried out following the Preferred Reporting Items for Systematic Reviews and Meta-Analyses (PRISMA) (Supplementary Tables S1 and S2) [23]. The review protocol was registered in PROSPERO with the reference number CRD42024556132. The review question focuses on eco-anxiety in the general population worldwide, in order to assess the presence of eco-anxiety in the general population and its possible differences among population groups.

The selection procedure was based on the “PICOS” Framework, as reported in Table 1.

Four electronic databases (PubMed, Scopus, Web of Science and PsycINFO) were then scrutinized using the following search string to find as many relevant articles as possible: “eco-anxiety” OR (“eco” AND “anxiety”) OR “climate anxiety” OR “climate worry” OR “solastalgia” OR “environmental distress” OR “eco distress” OR “eco-paralysis”. All databases were searched by title, abstract, and MeSH terms and keywords.

### 2.2. Inclusion/Exclusion Criteria

Articles were deemed eligible if they reported the eco-anxiety level resulting from cross-sectional studies performed on the general population. Other types of studies, such as reviews, meta-analyses, case studies, qualitative investigations, book chapters, editorials, and commentary studies, were not considered. When pertinent, these other types of publications were examined to identify further articles in their references. We included items published in English and Italian, from the beginning of each database until 28 August 2025. Studies including the assessment of different fields other than eco-anxiety or assessing eco-anxiety through non-validated tools were excluded. For population and comparison, no exclusion criteria were adopted.

### 2.3. Study Selection

The titles and abstracts obtained from the three databases were imported into the reference management software Zotero (version 6.0.37), which was used for the initial assessment of relevance. Subsequently, the next phase involved a title and abstract screening, where potentially suitable studies were independently reviewed by three authors (A.D.G., E.M., and F.Gr.). Following this, the full texts of these studies were independently examined by the same authors, and a subsequent discussion took place regarding their potential inclusion in the review. Any disagreements were resolved through consensus among the authors. All the steps were supervised by three other investigators (C.P., F.Ga., and F.V.).

### 2.4. Data Extraction

The collected data were organized into a table that presented bibliographic details (including author, year of publication, country), sample size, study participant/population with age and gender, together with the scale or questionnaire used to assess eco-anxiety, eco-anxiety level values, and significantly correlated variables when reported.

### 2.5. Study Quality and Evaluation

The quality assessment of the selected articles was conducted using the Newcastle–Ottawa Quality Assessment Scale, adapted from cohort and case–control studies to perform a quality assessment for cross-sectional studies, as previously described [24]. The quality of each study was individually scored by three authors (A.D.G., E.M., and F.Gr.), and any discrepancies were resolved through consensus among all the authors. The ultimate rating for each article was calculated as the average of the three authors’ scores.

### 2.6. Data Synthesis and Meta-Analysis

Only studies assessing eco-anxiety with validated and standardized instruments were considered for the quantitative synthesis. In particular, eligible tools included the Climate Anxiety Scale (CAS) and its validated derivatives (e.g., Climate Change Anxiety Scale—CCAS, Climate Change Anxiety Scale for Women’s Health—CCASWH, and other local adaptations), the Hogg Eco-Anxiety Scale (HEAS), and the Eco-Anxiety Questionnaire (EAQ). The first is a 13-item scale developed by Clayton and Karazsia in the United States, which considers two factors of the climate anxiety response: the cognitive-emotional impairments (such as difficulties concentrating and sleeping) and the functional impairments (such as difficulties socializing, working and studying) [16]. The HEAS, originally developed by Hogg et al. in Australia and New Zealand, is a 13-item questionnaire that looks for affective (e.g., feeling anxious), ruminative (e.g., persistent thoughts), and behavioural (e.g., difficulties working) symptoms of eco-anxiety and anxiety about the individual’s personal impact on the planet [21]. The EAQ, developed by Àgoston et al. in 2022, evaluates through 22 questions habitual ecological worry and the negative consequences of eco-anxiety [19]. When multiple studies from the same research group examined the same population cohort, only one of them was included in the meta-analysis, though the others were retained in the general overview.

For each study included in the meta-analysis, we extracted the following data: author, year, country, sample size (N), instrument, mean and standard deviation (SD) of eco-anxiety level. When only total scores were reported, values were normalized per item by dividing both mean and SD by the number of items (k), according to the original validation papers (CAS/CCAS/CCASWH/EMEA/HEAS = 13; EAQ = 22). This procedure enabled comparability across studies on their original Likert response range (e.g., 1–5 or 0–3).

When descriptive statistics were incomplete (e.g., data reported only as prevalence categories, ranges, or medians), we reconstructed mean and SD using established statistical methods for meta-analysis [25,26]. Studies for which no reliable reconstruction was feasible were excluded from quantitative pooling. To account for the heterogeneity of scoring ranges across instruments, we harmonized the data through transformation to a Percentage of Maximum Possible (POMP) scale [27]: POMP = [(Observed score − Minimum possible)/(Maximum possible − Minimum possible) × 100. This conversion standardizes scores to a 0–100 metric, interpretable as the percentage of the maximum possible eco-anxiety. Both means and SDs were transformed accordingly prior to pooling. Meta-analyses were performed in Jamovi (v. 2.5, MAJOR module), using raw means as effect size and applying random-effects models (DerSimonian–Laird and REML estimators, for meta-regression). Heterogeneity was evaluated with Cochran’s Q and I^2^ statistics, and 95% prediction intervals were calculated. Subgroup analyses were conducted by instrument type, while for the POMP dataset, a meta-regression including “instrument” as a moderator was carried out. Forest plots were generated with squares proportional to the inverse-variance weight of each study and horizontal lines indicating 95% confidence intervals.

## 3. Results

**Figure 1:**
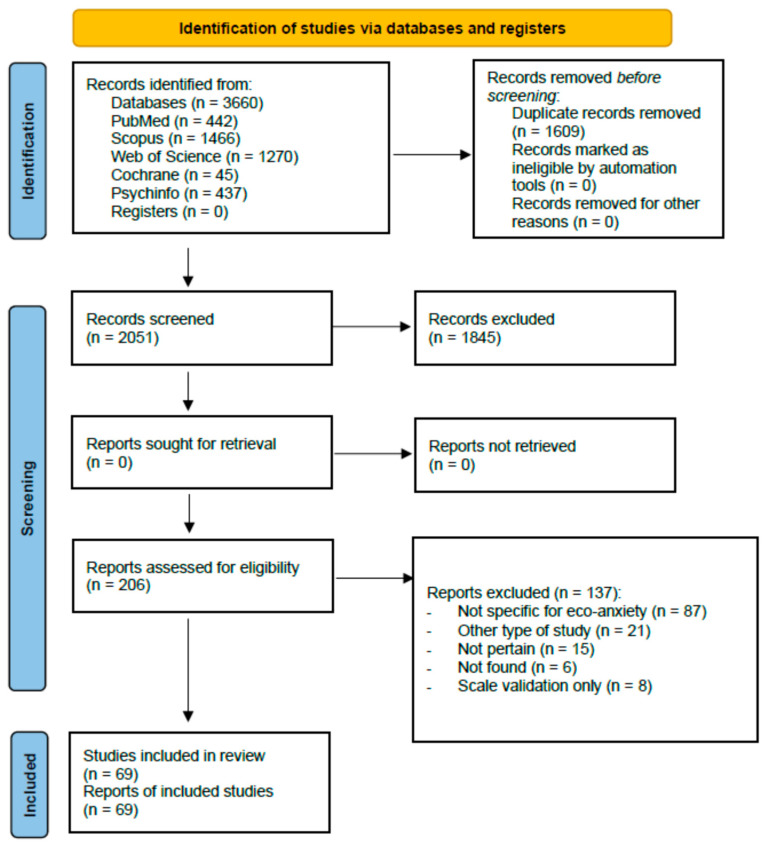
PRISMA flow chart of the review process.

On a total of 3660 articles found, 69 were considered eligible (Figure 1) [16,21,28,29,30,31,32,33,34,35,36,37,38,39,40,41,42,43,44,45,46,47,48,49,50,51,52,53,54,55,56,57,58,59,60,61,62,63,64,65,66,67,68,69,70,71,72,73,74,75,76,77,78,79,80,81,82,83,84,85,86,87,88,89,90,91,92,93,94].

Table 2 reports the main characteristics of the included studies.

The quality assessment with the Newcastle–Ottawa Scale (NOS) indicated that most studies scored in the fair-to-good range (5–8/10), while only seven [34,49,60,61,84,88,91] were rated as poor (≤4/10). The detailed quality evaluation is reported in Supplementary Table S3. High-quality studies typically combined large and representative samples with robust ascertainment and reporting, whereas lower-quality articles were mainly limited by small sample sizes, convenience recruitment, or incomplete reporting.

Articles were published between 2020 and 2025 and reported cross-sectional findings from diverse contexts. European contributions came from 38 studies [32,34,38,39,40,41,42,44,45,46,47,48,51,52,53,54,55,57,62,64,65,66,67,68,74,76,77,78,80,82,84,88,89,90,91,92,93]. Oceania was represented in 6 studies [21,33,49,50,59,72]. Americas’ populations were examined in four studies [16,61,69,70]. Asian countries were analyzed in 15 studies [31,35,36,37,43,56,58,60,71,73,79,83,85,86,94]. In Africa, eco-anxiety was assessed in three studies [28,29,30]. There were three studies involving countries from more than one continent [63,81,87].

Sample sizes varied widely—from small convenience samples (40 participants) [88] to large multi-country surveys (4000 individuals) [87]. Both males and females were represented, often with a female majority; age groups ranged from adolescents to older adults, with many studies focusing on young adults. As expected, measures of eco-/climate anxiety varied across the studies. The mainly used tools to assess eco-anxiety were the Climate Anxiety Scale (CAS; Clayton & Karazsia) [16,21,28,29,30,36,37,38,39,40,41,42,43,44,45,52,53,55,56,57,60,61,65,68,70,77,78,79,83,85,87,89,91,92,93], and the Hogg Eco-Anxiety Scale (HEAS) [21,31,36,46,47,48,49,50,51,52,58,59,61,62,64,67,69,71,72,73,75,76,80,81,82,84,86,88,90,94] in their different versions.

Research on eco-anxiety has expanded across regions, showing both shared patterns and context-specific nuances. In Europe, several studies linked climate anxiety to loneliness, mental health, perceived longevity, field of study and trust in science [32,38,39,40,41,42,46,47,80], while others reported correlations with affect and resilience [32,34,52,53,57,58,60,71,74,75,76,78,82,87,88] or with depression and life satisfaction [62,64]. Beyond Europe, African studies connected anxiety to environmental literacy or residence characteristics [28,30]. Turkey contributed one of the richest datasets, including validation work and studies on identity, health, and diet [35,36,37,43,71,73,86,94]. In North America, Canadian and U.S. studies showed that eco-anxiety is linked to existential meaning, affect and media exposure [16,61,69,70]. Australia and New Zealand were central in instrument development and characterization of the correlations with anxiety, depression and stress [21,33,49,50,59,72].

After transforming scores to the Percentage of Maximum Possible (POMP), standardized mean values ranged from a minimum of 1/100 [29] to a maximum of 82.5/100 [43] across the included studies (Table S4). Most estimates, however, clustered around the moderate range (40–50/100), indicating that while some populations reported very low or very high levels of eco-anxiety, the overall distribution suggested a central tendency toward moderate intensity [28,30,32,34,43,46,47,48,53,54,55,56,57,61,62,63,66,70,76,77,78,80,82,85,87,88]. Consistently, the random-effects pooled mean was ~34/100 (95% CI: 29–40). The broad confidence intervals for several studies further suggest considerable between-study heterogeneity.

As for the variables analyzed with eco-anxiety, gender differences are reported in 25 studies [16,21,28,31,36,43,44,50,54,55,57,58,64,65,67,73,74,80,81,84,86,87,88,93], with women showing quite always higher eco-anxiety or eco-anxiety subdomains than males. Age was the second most frequently studied variable, with 19 articles reporting higher levels of eco-anxiety in some age group, most frequently in the youngest [16,38,39,40,41,42,44,55,57,64,65,67,74,80,81,84,87,92]. Furthermore, the selected studies reported a wide variety of other variables correlated with eco-anxiety, such as psychological traits, place of residence, and engagement in pro-environmental behaviors, whose definition was very different across the studies and did not allow a comparison.

**Figure 2:**
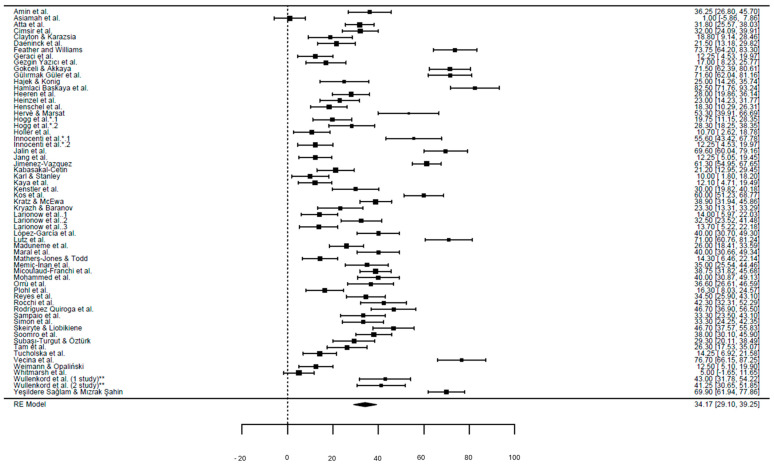
Forest plot of the meta-analysis (k = 60 studies). Horizontal bars indicate the 95% confidence intervals of individual effect sizes, while the diamond at the bottom represents the pooled estimate (random-effects model, τ^2^ = 388.35, I^2^ = 95.3%; *p* < 0.001). * Analyses derived from the same cohorts using different scales (CAS and HEAS); ** Studies reporting two distinct study designs [16,21,28,29,30,31,32,33,34,35,36,37,38,43,44,46,47,48,49,51,52,53,55,56,57,58,59,60,61,62,63,64,65,66,67,68,69,70,71,72,73,74,75,76,78,79,80,81,82,83,84,85,86,87,89,90,91,92,93,94].

Therefore, we performed the meta-analysis including only the 60 studies [16,21,28,29,30,31,32,33,34,35,36,37,38,43,44,46,47,48,49,51,52,53,55,56,57,58,59,60,61,62,63,64,65,66,67,68,69,70,71,72,73,74,75,76,78,79,80,81,82,83,84,85,86,87,89,90,91,92,93,94] that assessed eco-anxiety using validated and standardized instruments (CAS, CCAS and derivatives, HEAS, EAQ), for a total sample size exceeding 65,000 participants across different countries and cultural contexts (Table S4). After transforming all scores to the Percentage of Maximum Possible (POMP) to harmonize scales with different ranges, the overall pooled mean eco-anxiety level was approximately 34.2/100 (95% CI: 29.1–39.2), corresponding to a moderate level of eco-anxiety. Between-study heterogeneity was very high (I^2^ > 95.6%), reflecting differences in instruments, populations, and study settings (Figure 2). The prediction interval was 13–55/100, indicating that future similar studies may observe eco-anxiety levels ranging from very low to moderately high. Subgroup analysis showed that eco-anxiety scores varied depending on the instrument used. Studies employing the CAS or its derivatives (n = 34) consistently reported mean values of about 40/100 (95% CI: 36–44), representing the majority of available data. The HEAS (n = 26) tended to produce slightly higher estimates, averaging 46/100 (95% CI: 41–52), with a stronger emphasis on affective and behavioral components. The EAQ (n = 2) yielded the highest values at approximately 51/100 (95% CI: 47–55). These differences were statistically significant, indicating that the choice of measurement tool explained part of the heterogeneity observed across studies.

Meta-regression analyses further explored potential sources of heterogeneity among the examined variables—i.e., gender, age, year of publication and country. When modeling country as three macro-areas, studies from Western/Northern Europe showed lower eco-anxiety than Mediterranean/Eastern countries (β = −11.4 POMP; 95% CI: −23.5 to 0.6; *p* = 0.063), and Non-European samples were even lower (β = −18.1 POMP; 95% CI: −34.6 to −1.6; *p* = 0.033). This supports a gradient whereby Mediterranean/Eastern contexts exhibit the highest mean scores, followed by Western/Northern Europe and then Non-European samples. The association with publication year is non-significant (β = +1.5 POMP per year; 95% CI: −2.7 to 5.7; *p* = 0.48). These differences, although indicative, should be interpreted cautiously due to the heterogeneity in sample characteristics (students, workers, general population) and cultural as well as environmental contexts. However, these findings highlight the robustness of eco-anxiety as a measurable construct across diverse populations and contexts, while also emphasizing the role of methodological and cultural factors in shaping the magnitude of observed effects. The pooled estimates, along with the prediction interval, provide a reliable benchmark for future studies and for policymakers interested in monitoring eco-anxiety as an emerging public health concern, especially among some populations.

Several studies reported sex-disaggregated data, consistently indicating higher scores among women compared with men. Across the 15 studies reporting such data, the standardized mean difference (SMD) between women and men ranged from 0.15 to 0.45, corresponding to approximately 4–9 POMP points. On average, the difference was 0.30 SMD (6 POMP points; women ≈44/100; men 38/100; Q_between, *p* < 0.05). The meta-regression (REML) revealed no significant association between mean age and climate anxiety (β = −0.37 per year; 95% CI: −0.82 to +0.08; *p* = 0.11), indicating only a trend to report higher scores for younger groups. When the country was modeled as a predictor, Western/Northern European samples scored 11 POMP points lower than Mediterranean/Eastern countries (*p* = 0.06), and Non-European samples 18 points lower (*p* = 0.03). Excluding studies rated as lower quality (NOS = Poor) did not materially change the pooled estimates (pooled mean 34/100), supporting the robustness of the results.

Finally, sample size was not associated with effect size, and incomplete demographic reporting prevented a reliable multivariable assessment including age and sex simultaneously across all studies.

## 4. Discussion

A high variability emerged from the analysis of the available literature on eco-anxiety. Though our meta-analysis approach considered only those studies that were based on validated tools, it was found that the use of different assessment methods accounts for a great part of the heterogeneity found among the selected studies. This confirms the previously acknowledged gaps in eco-anxiety characterization and highlights the need for standardized research in this field [10].

The most significant focus of this research is the possible role of demographic variables such as gender and age in defining the way individuals experience eco-anxiety. Notably, the evidence suggests a gendered dimension for eco-anxiety, with the majority of the studies that examined gender as a variable identifying a significant association with females. In this regard, the literature shows that anxiety and related disorders in general are roughly twice as prevalent in women compared to men, and this may explain the gender difference observed in our study as a reflection of broader gender disparities in general anxiety and depression incidence, mainly due to exposure to stressful life events and biological factors [95]. Furthermore, this finding is in line with those of a recent review of the literature, which related gender differences in eco-anxiety to physiological and socioeconomic aspects such as women’s anatomy and lower access to cooling and sanitation facilities worldwide [96].

Similarly, age emerged as a critical determinant, with younger individuals consistently exhibiting higher levels of eco-anxiety. This trend may reflect greater awareness or concern about future environmental conditions among younger generations, who are likely to experience potentially harmful medium- to long-term consequences of climate change more acutely than others [16,38,39,40,41,42,44,55,57,64,65,67,74,80,81,84,87,92].

Both gender and age were shown to be related to climate change, even in a recent meta-analysis examining 33 correlates from 94 studies [97]. However, it should be noted that in our meta-regression analysis, the relationship between eco-anxiety and gender was confirmed, while that with age was not significant. This suggests that other age-related variables, such as educational level or environmental awareness, can play a role as mediators or bias sources in this relationship. Further research is needed to explore these aspects in depth.

These findings underline the value of exploring the interplay between demographic variables and the psychological dimensions of eco-anxiety, as such an approach provides insights into how diverse populations perceive and emotionally respond to the environmental crisis. Understanding these complexities makes it possible to tailor interventions for specific groups but also opens the door to examining the broader psychological implications of eco-anxiety. For instance, climate change anxiety has been frequently linked to general anxiety and depression [16,33,46,54,55,62,64,66,72,74,78,86,93], suggesting that it may both overlap with and exacerbate existing mental health challenges. Eco-anxiety can manifest through cognitive, emotional, and behavioral responses, such as persistent worries, psychological distress, or sleep disturbances. Concerns about environmental issues can also disrupt individuals’ ability to participate in work, education, or personal relationships, leading to functional impairments [16]. While eco-anxiety may amplify emotional distress, it is also closely linked to increased engagement in pro-environmental behaviors, as higher levels of worry or anxiety about climate phenomena have been shown to correspond with a stronger commitment to actions aimed at mitigating its effects [10]. Anxiety is an adaptive mechanism that encompasses cognitive and affective dimensions, prompting problem-solving behaviors aimed at reducing perceived risks [12]. The dual nature of eco-anxiety is highlighted by its role as both a psychological burden and a potential motivator for constructive environmental action, pointing to the need for interventions that balance emotional support with empowerment to act [98,99,100].

Finally, when considering the meta-regression results regarding the geographical distribution of selected studies, the higher values registered in countries overlooking the Mediterranean Sea suggest that exposure to a mild climate such as that of the Mediterranean Basin may increase the probability of developing eco-anxiety [96]. This factor, together with cultural and social differences among countries, should be studied in depth as a possible predictor.

However, some limitations should be considered when interpreting these results. One significant limitation is the considerable variability across the selected studies in terms of populations, variables, and methodologies, which can influence how eco-anxiety is experienced and reported. A particularly challenging aspect was the inconsistency in the methods used to assess eco-anxiety, with studies relying on self-reported measures, and employing different scales or questionnaires. This lack of standardization in assessment tools introduces variability in how eco-anxiety is defined, measured, and reported, limiting the ability to aggregate the findings meaningfully. We have tried to overcome this issue by performing a meta-regression analysis. Furthermore, due to its aim, our review considered only cross-sectional studies; analyzing longitudinal studies could better contribute to characterizing the determinants of eco-anxiety. In addition, we included articles published in the English or Italian language, and we did not consider the grey literature in our search; this could have generated selection biases, maybe overlooking the presence of eco-anxiety in some populations worldwide. Considering that significant geographical differences emerged from the meta-regression analysis, this aspect should be addressed in future studies.

Nevertheless, our findings highlighted some common aspects that should be considered when planning policies aimed at mitigating the health effects of climate change. These include the importance of recognizing the cognitive and emotional responses to environmental worsening, such as eco-anxiety, as well as the need for targeted interventions that consider demographic factors like people’s gender and residence. By focusing on these aspects, effective strategies aimed at addressing environmental concerns while supporting mental well-being and encouraging pro-environmental behaviors could be created.

## 5. Conclusions

With the climate crisis becoming more urgent, eco-anxiety represents a growing phenomenon that needs to be considered by public health authorities. Evidence regarding the dimensions and correlates of eco-anxiety shows great variability. Therefore, more specific and homogeneous research in this field is needed.

However, the findings of this review highlight the importance of considering demographic factors in understanding eco-anxiety, as they can highlight how different groups perceive and react to the environmental crisis, revealing the more vulnerable categories to which interventions should be primarily addressed. In particular, the meta-analysis of the literature shows that eco-anxiety affects women more than men and some countries more than others. Governments, especially those of the most interested countries, should then tackle this issue by identifying the most appropriate methods and settings to communicate risks and enhance people’s resilience to environmental challenges.

## Figures and Tables

**Table 1:** “PICOS” Framework used for the study selection procedures.

PICO Element	Keywords
P = Patient, problem or population	General population
I = Intervention	Assessment of eco-anxiety through validated methods
C = Comparison; control or comparator	Age-, gender- and condition-matched control group (if present)
O = Outcome(s)	Estimate of eco-anxiety level in the general population with possible differences among population groups and, if available, correlated variables
S = Study type	Cross-sectional studies

**Table 2:** Main characteristics of the included studies.

[Ref]	Author Year Country	Sample Characteristics	Scale/Questionnaire	Main Results	Newcastle-Ottawa Scale
[28]	Amin 2024 Egypt	620 undergraduate nursing students; 28.9% aged ≤20, 55.6% 20–22, 15.5% ≥22; Females 71.9%	CAS	Mean CAS score 31.9 ± 12.1. Females had higher climate anxiety (*p* = 0.013), while higher environmental literacy decreased climate anxiety (*p* = 0.010).	Good
[29]	Asiamah et al. 2025 Ghana	3994; ≥50 years; Females 47%	CAS	Mild 46%, moderate 40%, severe 14%.	Fair
[30]	Atta et al. 2024 Egypt	359 university nursing staff/colleagues; largest age group 30–<40 years = 49.3%; Females 77.4%	CAS	29.54 ± 5.26. Demographics did not influence climate anxiety. Geographical variables were significantly correlated with eco-anxiety (*p* < 0.001).	Good
[31]	Cimsir et al. 2024 Turkey	445; 29.76 years (18–65 years); Females 64.3%	HEAS	Females (M = 0.98, SD = 0.55) had significantly higher scores than males (M = 0.82, SD = 0.63).	Fair
[16]	Clayton and Karazsia 2020 USA	197; ≅50% aged 25–34 years (18–>70); Females 40.6%	CAS	Study 1. Cognitive impairment 1.75 ± 0.97, Functional impairment 2.09 ± 1.08, Experience 3.08 ± 1.20, Behavioral Engagement 3.67 ± 0.84, Environmental Identity 3.11 ± 0.97, Negative Emotionality 2.30 ± 0.96, Depression/Anxiety 2.04 ± 1.06. Women scored significantly higher than men in behavioral engagement (*p* = 0.007). The youngest age groups (18–35) scored higher than the other age groups in cognitive impairment and in functional impairment (*p* < 0.001).	Fair
[32]	Daeninck et al. 2023 UK	473 university students in environmental and non-environmental courses; 24.49 ± 6.10 years; Females 53.7%	CAS	Environmental group was more climate anxious (M = 26.79, SD = 10.33) than the non-environmental group (M = 21.28, SD = 7.94, *p* < 0.001).	Fair
[33]	Feather and Williams 2021 New Zealand	771; 33 ± 11.85 years; Females 48%	CCAS	Mean CAS score 1.62 ± 0.62. The correlations between psychological flexibility, climate change anxiety, anxiety and depression were all negative and significant (*p* < 0.001), while the correlations between psychological inflexibility and climate change anxiety, anxiety and depression were all positive and significant (*p* < 0.001).	Fair
[34]	Geraci et al. 2024 Italy	224; 21.04 ± 1.65 (19–26); Females 61%.	CCAS	Mean CCAS score 1.49 ± 0.62; no significant correlation with age and gender; significant correlations with climate change awareness (r = 0.14, *p* < 0.05) and worry (r = 0.55, *p* < 0.01).	Poor
[35]	Gezgin Yazıcı et al. 2025 Turkey	664; 71.49 ± 6.21 years; Females 54.4%	CCAS	Mean CCAS score 1.68 ± 0.80; correlation with Insomnia Severity Index (r = 0.26, *p* < 0.001).	Fair
[36]	Gokceli & Akkaya 2025 Turkey	406 university students; 85.5% aged 17–21 years; 12.1% 22–26 years; Females 79.3%	HEAS	Mean HEAS score 27.90 ± 8.44 in females, 26.94 ± 10.03 in males (*p* = 0.386); correlation with program of study (*p* = 0.007) and with nature relatedness levels (r = 0.160, *p* = 0.001).	Fair
[37]	Gülırmak Güler et al. 2024 Turkey	321 nursing students; 20.4 ± 2.61 years; Females 81%	CCAS	Mean CCAS score 50.2 ± 12.4; association with future anxiety (R = 0.234, *p* = 0.000) and intolerance of uncertainty (r = 0.562, *p* < 0.001).	Fair
[38]	Hajek and Konig 2022 Germany	3091; 46.5 ± 15.3 years (18–74); Females 49.5%	CAS	The average level of climate anxiety was 2.0 (SD = 1.2). Association with higher loneliness (β = 0.06, *p* < 0.001) and with higher perceived social isolation (β = 0.10, *p* < 0.001) in the total sample and among individuals aged 18 to 29 years, 30 to 49 years, and 50 to 64 years (*p* < 0.01) but not in those aged 65 to 74 years.	Good
[39]	Hajek and Konig 2022 Germany	3091; 18–74 years; upper secondary school 39.9%; Full-time employed 44.2%; 45.9% with chronic disease	CAS	The average level of climate anxiety was 2.0 (SD = 1.2). Climate anxiety was higher among younger (β = −0.005, *p* < 0.001) and full-time employed (β = 0.07, *p* < 0.01) individuals, individuals without chronic conditions (β = −0.08, *p* < 0.001).	Good
[40]	Hajek and Konig 2023 Germany	3015; 46.5 ± 15.3 years (18–74); Females 49.9%	CAS	The average level of climate anxiety was 2.0 (SD = 1.2). Association with lower perceived longevity among the total sample (β = −1.41, *p* < 0.01) and among individuals aged 18 to 29 years (β = −3.58, *p* = 0.01), but not in the other age groups.	Good
[41]	Hajek and Konig 2023 Germany	3091; 46.5 ± 15.3 years (18–74); Females 49.5%.	CAS	The average level of climate anxiety was 2.0 (SD = 1.2). Correlations with age (r = −0.18, *p* < 0.001), depressive (r = −0.30, *p* < 0.001) and anxiety (r = −0.31, *p* < 0.001) symptoms.	Good
[42]	Hajek and Konig 2024 Germany	3091; 46.5 ± 15.3 years (18–74); Females 49.5%.	CAS	The average level of climate anxiety was 2.0 (SD = 1.2). A greater belief in science was significantly associated with higher (log) climate anxiety (β = 0.003, *p* < 0.001), mainly in young adults aged 18–29 years (β = 0.006, *p* < 0.001).	Good
[43]	Hamlaci Baskaya et al. 2024 Turkey	978; 27.87 ± 6.82 years (18–49); Females 100%	CCASWH	Mean CCASWH score 55.89 ± 17.12. Lower values in women who do not think that there is environmental pollution in their region (β = −0.132, *p* = 0.006) and in participants without allergies (β = −0.088, *p* = 0.021).	Fair
[44]	Heeren et al. 2022 Belgium	2080; 43.04 ± 13.52 years (17–84); Females (51.88%); 2034 (97.8%) from Europe, 46 (2.2%) from Africa.	CAS	11.64% of participants experienced climate anxiety more often than “sometimes”. CAS in Females was M = 2.12 (SD 0.69) and in Males M = 1.99 (SD 0.70) (*p* < 0.001). Correlations with age (r = −0.15), cognitive-emotional (r = 0.94) and functional (r = 0.90) impairments, experience of climate change (r = 0.35).	Good
[45]	Heeren et al. 2023 Belgium	874; 38.42 ± 14.11 years (18–81); Females 51.37%; 52.40% from France, 44.16% from Belgium, 2.17% from Switzerland	CCAS	Mean cognitive-emotional component score 16.46 ± 5.75, mean functional component score 11.22 ± 4.34.	Fair
[46]	Heinzel et al. 2023 Germany	486; 29.43 ± 10.63 years (18–73); Females 75.1%	HEAS	Mean affective symptoms subscale score 0.69 ± 0.60, rumination 0.60 ± 0.67, behavioral symptoms 0.33 ± 0.50, anxiety about personal impact 1.20 ± 0.70.	Fair
[47]	Henschel et al. 2025 Germany	322; 36.6 ± 14.8; Females 67.4%	HEAS	Mean HEAS score 0.55 ± 0.50; Subscale Affective Symptoms 0.53 ± 0.59; Subscale Rumination 0.39 ± 0.55; Subscale Behavioral Symptoms 0.33 ± 0.54; Subscale Personal Impact Anxiety 0.94 ± 0.80.	Fair
[48]	Hervè & Marsat 2023 France	671; 49.9 years; Females 50.7%	HEAS	Mean HEAS score 0.16; median 0.13 (0.04–0.23)	Fair
[49]	Hogg et al. 2021 Australia	334 undergraduates; 22.23 ± 6.65 years; Females 59%	HEAS	65.8% experienced eco-anxiety. All the HEAS domains correlated with anxiety and depression (*p* < 0.01).	Poor
[21]	Hogg et al. 2023 Australia	530; 39.5 ± 16.5 years (18–86); Females 63.2%	HEAS, CAS	Mean HEAS subscale scores: affective symptoms Females 0.91 ± 0.80, Males 0.71 ± 0.75; rumination Females 1.09 ± 0.89, Males 0.93 ± 0.94; behavioral symptoms Females 0.58 ± 0.72, Males 0.51 ± 0.69; personal impact anxiety Females 1.47 ± 0.89, Males 1.04 ± 0.91; Mean CAS subscale scores: cognitive-emotional impairments Females 1.73 ± 0.71, Males 1.66 ± 0.67; functional impairments Females 1.94 ± 0.84, Males 1.82 ± 0.89, no significant gender differences.	Fair
[50]	Hogg et al. 2024 Australia	530; 39.5 ± 16.5 years (18–86); Females 63.2%	HEAS	Mean HEAS subscale scores: affective symptoms 0.85 ± 0.79; rumination 1.05 ± 0.91; behavioral symptoms 0.56 ± 0.71; personal impact anxiety 1.35 ± 0.92. Correlated with more symptoms of generalised anxiety and depression, lower life satisfaction, and more pro-environmental behaviour and readiness to adopt a low carbon lifestyle (*p* < 0.01). Age was associated with more rumination (*p* < 0.01) and less personal impact anxiety (*p* < 0.05), and females experienced more affective symptoms (*p* < 0.05) and personal impact anxiety (*p* < 0.001) than males.	Fair
[51]	Holler et al. 2025 Iceland	47; 54.0 ± 16.7 years (20–92); Females 57.4%	HEAS	Mean HEAS score 4.19 ± 6.65. Correlation with worries about air pollution from studded tires.	Fair
[52]	Innocenti et al. 2023 Italy	150; 34.14 ± 11.07 years; Females 52.7%	HEAS, CCAS	Mean HEAS score 21.74 ± 15.08; CCAS cognitive impairment score was 11.80 ± 7.55; CAS cognitive functional impairment score 8.54 ± 6.62; correlations with Climate Change Worry Scale, Eco-Paralysis Scale (*p* < 0.001).	Fair
[53]	Innocenti et al. 2023 Italy	394; 33.1 ± 11.8 years; Females 64.2%	CCAS	Mean CCAS cognitive impairment score was 11.59 ± 3.47, *p* = 0.344; cognitive functional impairment score 6.23 ± 1.94; negative correlations with Global Self-Efficacy score (*p* < 0.01).	Fair
[55]	Jalin et al. 2024 France	522	EMEA	Mean EMEA total score 46 ± 12.5. Association with female gender (*p* < 0.05), level of education (*p* < 0.01), not having children (*p* < 0.001), exposure to media and connectedness to nature (*p* < 0.001), depression and anxiety (*p* < 0.05), negative outcomes, obstruction and coercive affects of the environmental trait affect questionnaire (*p* < 0.001).	Fair
[54]	Jalin et al. 2025 France	262; 48 ± 14.2 years (19–92); Females 68%	EMEA; CAS	Mean EMEA total score 49.2 ± 12.4; Mean CAS total score 51.2 ± 12.3. Higher EMEA total score, anxiety-depressive manifestations and relational disturbances (*p* = 0.001) in young people; higher anxiety-depressive manifestations (*p* = 0.019) and relational disturbances (*p* = 0.024) in women; lower total score in participants with children (*p* = 0.001).	Fair
[56]	Jang et al. 2023 Korea	459; 44.2 ± 13.5; Females 51%	CCAS	Mean total score 1.49 ± 0.54.	Fair
[57]	Jimenez-Vazquez 2025 Spain	1065; 14.0 ± 1.49 years (12–18); Females 49%	CAS	Mean CAS total score 16.19 ± 5.44. Higher levels in younger (*p* = 0.014), female (*p* = 0.003), and low-vs-high socioeconomic status participants (*p* = 0.007).	Fair
[58]	Kabasakal-Cetin 2023 Turkey	605 undergraduates; Males 21.6 ± 1.8 years, Females 20.8 ± 1.8 years; Females 61.2%	HEAS	Eco-anxiety scale total score was higher in female (12.2 ± 6.9) than in male students (14.0 ± 8.0) (*p* = 0.003). Correlations with healthy and balanced nutrition (r = −0.124, *p* < 0.05) low fat diet (r = −0.091, *p* < 0.05), meat reduction (r = 0.116, *p* < 0.05) and local food (r = 0.113, *p* < 0.05), environmental awareness (r = 0.176, *p* < 0.001) and reusability (r = 0.094, *p* < 0.05).	Fair
[59]	Karl & Stanley 2024 Australia	287; 35.0 ± 12.3 years; Females 48.4%	HCAS	Mean HCAS subscale scores Affective symptoms 0.6 ± 0.7, ruminative symptoms 0.3 ± 0.5, behavioral symptoms 0.4 ± 0.7, personal impact anxiety 0.6 ± 0.7; correlations with domains of the Comprehensive Inventory of Mindfulness Experiences and solastalgia (*p* < 0.05).	Fair
[60]	Kaya et al. 2025 Turkey	1126 pregnant women; 28.4 ± 5.6 years (18–44); Females 100%	CAS	Baseline Mean CAS total score 19.3 ± 7.4.	Poor
[61]	Kenstler USA 2025	169 (36 environmental sciences majors—ES and 133 nonenvironmental science majors—NES); 20.0 ± 1.8 years; Females 57.4%	HEAS, CAS	Mean HEAS total score ES 0.90 ± 0.81, NES 0.69 ± 0.68 (*p* = 0.06); mean CAS total score ES 1.60 ± 0.77, NES 1.39 ± 0.59 (*p* < 0.05). Positive correlations with pro-environmental behaviors (*p* < 0.001).	Poor
[62]	Kos et al. 2025 Slovenia	324; 23.8 ± 3.8 years (18–30); Females 70.4%	HEAS	Mean HEAS total score 1.8 ± 0.6. Positive correlation with anxiety and depression and negative correlation with reproductive wish (*p* < 0.001).	Fair
[63]	Kratz & McEwan 2025 Africa, Europe, Americas, Asia	151; 47 ± 12.9 years (19–82); Females 67%	EAQ	Mean EAQ score 56.2 ± 11.1. Positive correlations with pro-environmental behaviors and perceived landscape change (*p* < 0.01).	Fair
[64]	Kryazh and Baranov 2025 Ukraine	446; 32.3 ± 11.0 years (17–75), Females 67.9%	HEAS	Mean HEAS subscale scores: affective symptoms Females 0.70 ± 0.78, Males 0.54 ± 0.73; rumination Females 0.57 ± 0.68, Males 0.48 ± 0.63; behavioral symptoms Females 0.52 ± 0.70, Males 0.54 ± 0.73; personal impact anxiety Females 0.59 ± 0.67, Males 0.45 ± 0.66, not significant. Younger participants showed higher personal impact anxiety (*p* = 0.014). All eco-anxiety domains positively correlated with depression and pro-environmental behavior and negatively correlated with life satisfaction (*p* < 0.001).	Fair
[65]	Larionow et al. 2022 Poland	603; 25.32 ± 9.59 (18–70); Females 57%	CAS	Total mean CAS score 20.34 ± 8.68. Higher levels in females (*p* < 0.001). Negative correlations with age (*p* = 0.006) and education (*p* < 0.001).	Good
[66]	Larionow et al. 2024 Poland	420; 26.2 ± 10.6 (18–70); Females 82.4%	CCWS	Total mean CCWS score 23.02 ± 8.41; correlations with anxiety (*p* < 0.001) and depression (*p* < 0.01).	Good
[67]	Larionow et al. 2024 Poland	634; 28.1 ± 10.7 (18–67); Females 81.4%	HEAS	Mean HEAS subscale scores: Affective symptoms 0.47 ± 0.58, ruminative symptoms 0.37 ± 0.51, behavioral symptoms 0.34 ± 0.58, personal impact anxiety 0.47 ± 0.58. 80 people (12.62% of the total sample) had clinically significant levels of eco-anxiety. Higher levels of anxiety about their personal impact in females than in males (*p* < 0.001); negative correlations with age and education (*p* < 0.05).	Good
[68]	López-García et al. 2025 Spain	308 young adults; 24.56 ± 3.69 (18–30); Females 51.9%	CCAS	The mean value for eco-anxiety (M = 2.564 ± 0.928) resulted very low.	Fair
[69]	Lutz et al. 2023 Canada	132; 21.2 ± 4.9 years (17–50); Females 72.6%	HEAS	Fairly low levels of eco-anxiety, with the mean being below the scale midpoint (2.13 on a 1–5 scale). Eco-anxiety was negatively associated with positive deactivated affect (r = −0.18, *p* = 0.047) and positively associated with both negative activated (r = 0.26, *p* = 0.004) and deactivated affect (r = 0.22, *p* = 0.012).	Good
[70]	Maduneme et al. 2024 USA	398; 20 years; Females 67%	CCAS	Mean CCAS score 2.04 ± 0.60. Media exposure variables explained approximately 33% of the variance in climate anxiety, with the frequency of media use and attention given to climate change news significantly predicting climate anxiety (*p* < 0.001).	Good
[71]	Maral et al. 2025 Turkey	392; 27.71 ± 6.71 years (18–59); Females 71.7%	HEAS	Mean HEAS score 15.86 ± 8.82. Correlation with mental wellbeing (*p* < 0.05).	Good
[72]	Mathers-Jones & Todd 2023 Australia	96; 20.9 ± 3.4; Females 70.8%	HEAS	Mean HEAS score 0.43 ± 0.48. Correlations with anxiety, depression and stress (*p* < 0.01).	Fair
[73]	Memiç-İnan et al. 2025 Turkey	736 young adults; 20.9 ± 1.8 years; Females 70.5%	HEAS	Mean HEAS score 13.4 ± 5.9. Higher eco-anxiety scores were reported among females and those studying in health-related departments (*p* < 0.05).	Good
[74]	Micoulaud-Franchi et al. 2023 France	1004; 43.47 years ± 13.41, (19–66); Females 54.1%	EAQ	EAQ mean total score Males 48.37 ± 13.22, Females 50.52 ± 12.41 (*p* = 0.008); higher in participants aged <35 years (*p* < 0.001). Correlation with anxiety (*p* < 0.001) and depression (*p* = 0.011).	Good
[75]	Mohammed 2025 Iraq	385; 29.50 ± 13.91 years; Females 58.5%; urban 64.5%, suburban 26.9%, rural 8.5%	HEAS	Eco-anxiety levels resulted mild 38%, moderate 43%, severe 19%. Significant associations was found between the level of eco-anxiety and the type of residence (χ^2^, *p* = 0.021) and city (χ^2^, *p* = 0.006).	Fair
[76]	Orrù et al. 2024 Italy	351 adults; 31 years (18–74); Females 66.7%	HEAS	Worry and emotion dysregulation were significant positive predictors of eco-anxiety; older age predicted lower eco-anxiety.	Good
[77]	Parmentier et al. 2023 France	431; 37.6 ± 14.6 (18–78); Females 71.6%	CCAS	Cognitive-emotional impairment (CEI) 1.84 (0.72), functional impairment (FI) 1.83 (0.81). Both factors of the CCAS exhibited positive and significant correlations with the environmental crisis perception scale (r = 0.39, *p* < 0.001 for CEI and r = 0.35, *p* < 0.001 for FI). Both eco-worry and trait anxiety significantly predicted CEI and FI (*p* < 0.001).	Fair
[78]	Plohl et al. 2023 Slovenia	442; 21.6 ± 1.7 (18–24); Females 75.8%	CAS; CCWS	Total mean CAS score 1.65 ± 0.71. Correlations with anxiety, stress, and climate worry (*p* < 0.001).	Good
[79]	Reyes et al. 2021 Philippines	433; 20.4 ± 1.6 years (18–26); Females 66.5%	CCAS	CCAS M = 2.38, SD = 0.77; MHI M = 127.71, SD = 24.42. Climate change anxiety and mental health are significantly negatively correlated (r = −0.37, *p* < 0.001). Climate change anxiety has a significant positive correlation with Psychological Distress (r = 0.39, *p* < 0.001), but no correlation with Psychological Wellbeing (r = −0.05, *p* = 0.140).	Fair
[80]	Rocchi et al. 2023 Italy	335; 32.06 ± 11.26 years (18–73); Females 61.8%	HEAS	Higher Affective Symptoms (M = 1.05, vs. M = 0.80, *p* = 0.005) and Anxiety about personal impact (M = 1.49, vs. M = 1.21, *p* = 0.006) were found in females than males. People aged <=30 reported significantly higher means than participants >30 in Affective Symptoms (M = 1.08 vs. M = 0.76, *p* = 0.005) and Anxiety about personal impact (M = 1.61 vs. M = 1.17, *p* < 0.001).	Good
[81]	Rodríguez Quiroga et al. 2024 Argentina and Spain	1538 (Argentina n = 990; Spain n = 548); Spain: Females 86%, 22.6 ± 6.1 years (16–57); Argentina: Females 56.8%; 40.8 ± 17.0 years (14–89)	HEAS	HEAS subscale scores: affective symptoms Females 0.89 ± 0.64, Males 0.72 ± 0.60 (*p* < 0.001); rumination Females 0.66 ± 0.58, Males 0.58 ± 0.58 (*p* < 0.01); behavioral symptoms Females 0.58 ± 0.63, Males 0.54 ± 0.58 (*p* = 0.218); personal impact anxiety Females 0.75 ± 0.64, Males 0.55 ± 0.61 (*p* < 0.001). Spanish participants had higher scores on the affective symptoms and personal impact anxiety factors respect to the Argentinian ones (*p* < 0.001). Younger participants tended to report higher scores on affective and behavioral symptoms and on personal impact (*p* < 0.001).	Good
[82]	Sampaio et al. 2023 Portugal	623 (F 81.5%); 20.46 ± 1.83 years	HEAS	The affective symptoms (2.86), rumination (1.52) and behavioural symptoms (1.60) subscale models were not significantly explained by any predictors (gender, age, schooling, living area, fathers’ and mothers’ school attainment). Only personal impact subscale (2.65) was significantly predicted by paternal education attainment (R^2^ = 0.026, *p* = 0.012, B = 0.878, *p* = 0.001).	Good
[83]	Simon et al. 2022 Philippines	452 university students; 19.18 ± 0.99 years	CCAS	Cognitive Emotional Impairment 18.61 ± 6.81; Functional Impairment 9.8 ± 9.8.	Fair
[84]	Skeiryte & Liobikiene 2025 Lithuania	705; 41.3 years; Females ≈55%	HEAS	Women reported higher eco-emotions than men—anxiety (t = 4.556, *p* < 0.001). Across age groups (17–24, 25–34, ≥35), differences were non-significant for anxiety (F = 1.059, *p* = 0.385).	Poor
[85]	Soomro et al. 2024 China	163 from flood-affected districts in Sindh and local schools/centres: 117 children (6–16 years) and 46 parents; children: 68 boys, 49 girls	CCAS	Climate-Change Anxiety (CCA) correlated with Climate-Change Education/Action (CCEA) (r = 0.524, *p* < 0.01) and with Mental Health (MH) outcomes (r = 0.513, *p* < 0.01); higher CCA and MH predicted higher Children Stress Index (β = 0.245 and 0.410, both *p* < 0.01), CCA predicted higher CCEA (β = 0.219, *p* < 0.05), and CCA together with CCEA predicted higher MH (β = 0.277, *p* < 0.01; β = 0.178, *p* < 0.05).	Fair
[86]	Subaşı-Turgut & Öztürk 2025 Turkey	367; 18–25 years; Females 49%	HEAS	Women reported higher eco-anxiety (*p* = 0.002). Eco-anxiety positively correlated with health anxiety and social maladjustment (*p* < 0.001).	Fair
[87]	Tam et al. 2023 China, India, Japan, USA	4000	CCAS	Total mean CCAS score China 2.223 ± 0.860, India 2.690 ± 0.856, Japan 1.644 ± 0.636, USA 1.637 ± 0.866. Higher levels in males from China and the U.S. (*p* < 0.001), lower in India (*p* < 0.05). Younger participants reported stronger climate change anxiety in India and the U.S., lower in China (*p* < 0.001). Positive correlation with income in China (*p* < 0.001) and negative in Japan (*p* < 0.01). Positive correlation with education in India (*p* < 0.01).	Good
[88]	Trifunović & Rajčević 2024 Bosnia and Herzegovina	40 geography teachers; Females 77.5%; 45 years	HEAS	HEAS-13 subscale means were Affective 0.625, Rumination 0.925 (highest), Behavioral impairment 0.583, Personal-impact anxiety 0.550; women scored higher than men on Affective (0.702 vs. 0.361), Rumination (1.043 vs. 0.519) and Personal-impact (0.677 vs. 0.111), while rural teachers exceeded urban on Rumination (1.500 vs. 0.781; z = 2.195), Behavioral impairment (0.958 vs. 0.490; z = 1.858) and Personal-impact (0.875 vs. 0.469; z = 3.740).	Poor
[89]	Tucholska et al. 2024 Poland	333 adults; Females 68.8%	CCAS	CCAS scores were low–to–moderate (Cognitive-Emotional Impairment M = 1.60, Functional Impairment M = 1.52 on a 1–5 scale) with modest climate emotions (e.g., fear M = 2.91, anxiety M = 2.73), and personality traits and time perspective emerged as key predictors of CCAS.	Fair
[90]	Vecina et al. 2025 Spain	1911 adults; 18–88 years	HEAS	Total mean HEAS score 2.3 ± 0.87. 51.8% of respondents exhibited mild eco-anxiety. Correlation with eco-worry (*p* < 0.001).	Fair
[91]	Weimann & Opaliński 2024 Poland	431; 23 ± 12 years (18–84) Females 71%	CAS	Mean CAS total score 1.51 ± 0.57.	Poor
[92]	Whitmarsh et al. 2022 UK	1338; ≈47.1 years; Females ~53%	CCAS	Very low mean climate anxiety (≈1.25/5). Higher among younger adults; small negative link with wellbeing and positive links with climate identity and self-reported PEB (Pro-Environmental Behavior).	Fair
[93]	Wullenkord et al. 2021 Germany	1011; 43.9 ± 13.9 (18–69); Females 51.1%	CAS	Mean total CAS score 1.71 ± 0.82. Higher climate anxiety in females (*p* = 0.003). Correlation with anxiety and depressiveness (*p* < 0.01).	Good
[94]	Yeşildere Sağlam & Mızrak Şahin 2025 Turkey	456 women (reproductive age); mean age 26.94 (SD 7.0).	HEAS	Mean score 27.28 ± 6.44; higher in women with pre-menstrual syndrome (PMS) (*p* < 0.001).	Fair

## Data Availability

No new data were created or analyzed in this study. Data sharing is not applicable to this article.

## Supplementary Materials

The following supporting information can be downloaded at: https://www.mdpi.com/article/10.3390/healthcare13212716/s1, Table S1: PRISMA checklist; Table S2: PRISMA abstract checklist; Table S3: NOS-based quality evaluation of the selected studies; Table S4: Data used in the meta-regression analysis.

## Abbreviations

The following abbreviations are used in this manuscript:

CAS	Climate Anxiety Scale
CCAS	Climate Change Anxiety Scale
CCAQ	Climate Change Anxiety Questionnaire
CCASWH	Climate Change Anxiety Scale for Women’s Health
CCWS	Climate Change Worry Scale
CSI	Children’s Stress Index (CSI)
DASS-21	Depression Anxiety Stress Scale
EAQ	Eco-Anxiety Questionnaire
EC	Environmental Crisis
EWS	Eco-Worry Scale
GAD-7-C	Generalised Anxiety Disorder scale anxiety
HEAS	Hogg Eco-Anxiety Scale
HINT	Habit Index of Negative Thinking
PRISMA	Preferred Reporting Items for Systematic Reviews and Meta-Analyses
