# Supplementary material for: Assessing the Presence of Eco-Anxiety in the General Population: A Systematic Review, Meta-Analysis and Meta-Regression
Source: Healthcare (Basel). 2025 Oct 27;13(21):2716. doi: 10.3390/healthcare13212716 (PMC12609564; doi:10.3390/healthcare13212716)
Supplement: Supplementary file 1 [file healthcare-13-02716-s001.zip › healthcare-3846057-supplementary.pdf]

Table S3. NOS-based quality evaluation of the selected studies

Ref	Author/Year	Country	N	Representativeness (0–2)	Sample Size (0–1)	Non Respondents (0–1)	Exposure Ascertainment (0–1)	Comparability_Total (0–2)	Outcome Assess (0–1)	Statistics (0–1)	Reporting (0–1)	NOS_Total (0–10)	Quality Score
28	Amin et al. (2024)	Egypt	620	1	1	0	1	1	1	1	1	8	Good
29	Asiamah et al. (2024)	Ghana	3994	1	0	0	0	1	1	1	1	6	Fair
30	Atta et al. (2024)	Egypt	359	1	1	0	1	1	1	1	1	8	Good
31	Cimsir et al. (2024)	Turkey	445	1	1	0	1	0	1	1	1	6	Fair
16	Clayton & Karazsia, 2020	USA	197	1	1	0	1	1	1	1	1	7	Fair
32	Daeninck et al. (2023)	UK	473	1	1	0	1	0	1	1	1	6	Fair

33	Feather & Williams (2021)	New Zealand	771	1	1	0	1	0	1	1	1	6	Fair
34	Geraci et al. (2024)	Italy	224	0	0	0	1	0	1	1	1	4	Poor
35	Gezgin Yazıcı et al. (2025)	Turkey	664	1	1	0	1	0	1	1	0	5	Fair
36	Gokceli & Akkaya (2025)	Turkey	406	0	1	0	1	1	1	0	1	5	Fair
37	Gülırmak Güler et al. (2024)	Turkey	321	0	1	0	1	0	1	0	0	3	Poor
38	Hajek & Konig (2022)	Germany	3091	1	1	0	1	1	1	0	0	5	Fair
39	Hajek & König (2022)	Germany	3091	1	1	1	1	2	1	1	1	9	Good
40	Hajek & Konig (2023)	Germany	3015	1	1	0	1	1	1	1	0	6	Fair
41	Hajek & Konig (2023)	Germany	3091	1	1	0	1	0	1	1	0	5	Fair

42	Hajek & Konig (2024)	Germany	3091	1	1	0	1	1	1	1	0	6	Fair
43	Hamlaci Baskaya et al. (2024)	Turkey	978	1	1	0	1	0	1	0	1	5	Fair
44	Heeren et al. (2022)	Belgium	2080	1	1	0	1	2	1	1	1	8	Good
45	Heeren et al. (2023)	Belgium/France/Switzerland	874	1	1	0	1	0	1	0	1	5	Fair
46	Heinzel et al. (2023)	Germany	486	1	1	0	1	0	1	0	1	5	Fair
47	Henschel et al. (2025)	Germany	322	1	1	0	1	0	1	0	1	5	Fair
48	Hervè & Marsat (2023)	France	671	1	1	0	1	0	1	0	1	5	Fair
49	Hogg et al. (2021)	Australia	334	0	1	0	1	0	1	1	0	4	Poor
21	Hogg et al. (2023)	Australia	530	1	1	0	1	1	1	0	1	6	Fair

50	Hogg et al. (2024)	Australia	530	1	1	0	1	1	1	0	0	5	Fair
51	Holler et al. (2025)	—	47	1	0	0	1	0	1	1	1	5	Fair
52	Innocenti et al. (2023)	Italy	150	1	0	0	1	0	1	1	1	5	Fair
53	Innocenti et al. (2023)	Italy	394	1	1	0	1	0	1	1	1	6	Fair
54	Jalin et al. (2024)	France	522	1	1	0	1	0	1	0	1	5	Fair
55	Jalin et al. (2025)	France	262	1	0	0	1	1	1	0	1	5	Fair
56	Jang et al. (2023)	Korea	459	1	1	0	1	0	1	0	1	5	Fair
57	Jimenez-Vazquez (2025)	Spain	1065	1	1	0	1	1	1	0	1	6	Fair
58	Kabasakal-Cetin (2023)	Turkey	605	1	1	0	1	1	1	1	0	6	Fair
59	Karl & Stanley (2024)	Australia	287	1	0	0	1	0	1	1	1	5	Fair

60	Kaya et al. (2025)	Turkey	1126	0	1	0	1	0	1	0	1	4	Poor
61	Kenstler (2025)	USA	169	0	0	0	1	0	1	1	1	4	Poor
62	Kos et al. (2025)	Slovenia	324	1	1	0	1	0	1	1	1	6	Fair
63	Kratz & McEwan (2025)		151	1	0	0	1	0	1	1	1	5	Fair
64	Kryazh & Baranov (2025)	Ukraine	446	1	1	0	1	1	1	1	1	7	Fair
65	Larionow et al. (2022)	Poland	603	2	1	0	1	1	1	1	1	9	Good
66	Larionow et al. (2022)	Poland	420	2	1	0	1	1	1	1	1	9	Good
67	Larionow et al. (2022)	Poland	634	2	1	0	1	2	1	1	1	10	Good
68	López-García et al. (2025)	Spain	308	1	1	0	1	0	1	1	1	6	Fair
69	Lutz et al. (2023)	Canada	132	1	0	1	1	2	1	1	1	8	Good

70	Madune me et al. (2024)	USA	398	1	1	0	1	2	1	1	1	8	Good
71	Maral et al. (2025)	Türkiye	392	1	1	0	1	1	1	1	1	7	Good
72	Mathers-Jones & Todd (2023)	Australia	96	1	0	0	1	1	1	1	1	6	Fair
73	Memiç-İnan et al. (2025)	Türkiye	736	1	1	0	1	1	1	1	1	8	Good
74	Micoulau d-Franchi et al. (2023)	France		1	1	0	1	1	1	1	1	8	Good
75	Mohamm ed et al., 2025	Iraq (Kurdistan Region)	385	1	1	0	1	0	1	1	1	6	Fair
76	Orrù et al. (2024)	Italy	351	1	1	0	1	1	1	1	1	7	Good
77	Parmenti er et al. (2023)	France	431	1	1	0	1	1	1	1	1	7	Fair

78	Plohl et al., 2023	Slovenia	442	1	1	0	1	1	1	1	1	8	Good
79	Reyes et al. (2021)	Philippines	433	1	1	0	1	1	1	1	1	7	Fair
80	Rocchi et al. (2023)	Italy	335	1	1	0	1	2	1	1	1	8	Good
81	Rodríguez Quiroga et al. (2024)	Argentina & Spain	1,538	1	1	0	1	1	1	1	1	8	Good
82	Sampaio et al. (2023)	Portugal	623	1	1	0	1	2	1	1	1	8	Good
83	Simon et al. (2022)	Philippines	452	1	1	0	1	0	1	1	1	6	Fair
84	Skeiryte & Liobikiene (2025)	Lithuania	705	1	1	0	0	0	0	1	0	4	Poor
85	Soomro et al. (2024)	China	163	1	0	0	1	0	1	1	1	5	Fair
86	Subaşı-Turgut & Öztürk (2025)	Turkey	367	1	1	0	1	0	1	1	1	6	Fair

87	Tam et al., 2023	China, India, Japan, USA	4000	2	1	0	1	2	1	1	1	9	Good
88	Trifunović & Rajčević (2024)	Bosnia and Herzegovina	40	0	0	0	1	0	1	1	1	4	Poor
89	Tucholska et al. (2024)	Poland	333	1	1	0	1	1	1	1	1	7	Fair
90	Vecina et al. (2025)	Spain	1911	1	1	0	1	1	1	1	1	7	Fair
91	Weimann & Opaliński (2024)	Poland	431	0	1	0	1	0	1	1	0	4	Poor
92	Whitmarsh et al. (2022)	UK	1338	1	1	0	1	1	1	1	1	7	Fair
93	Wullenkord et al., 2021	Germany	1011	2	1	0	1	1	1	1	1	9	Good
94	Yeşildere Sağlam & Mızrak Şahin (2025)	Turkey	456	1	1	0	1	1	1	1	1	7	Fair

Table S4. Data used in the meta-regression analysis

Ref	Author/Year	Country	N	Representativeness (0–2)	Sample Size (0–1)	Non Respondents (0–1)	Exposure Ascertainment (0–1)	Comparability_Total (0–2)	Outcome Assess (0–1)	Statistics (0–1)	Reporting (0–1)	NOS_Total (0–10)	Quality Score
28	Amin et al. (2024)	Egypt	620	1	1	0	1	1	1	1	1	8	Good
29	Asiamah et al. (2024)	Ghana	3994	1	0	0	0	1	1	1	1	6	Fair
30	Atta et al. (2024)	Egypt	359	1	1	0	1	1	1	1	1	8	Good
31	Cimsir et al. (2024)	Turkey	445	1	1	0	1	0	1	1	1	6	Fair
16	Clayton & Karazsia, 2020	USA	197	1	1	0	1	1	1	1	1	7	Fair
32	Daeninck et al. (2023)	UK	473	1	1	0	1	0	1	1	1	6	Fair

33	Feather & Williams (2021)	New Zealand	771	1	1	0	1	0	1	1	1	6	Fair
34	Geraci et al. (2024)	Italy	224	0	0	0	1	0	1	1	1	4	Poor
35	Gezgin Yazıcı et al. (2025)	Turkey	664	1	1	0	1	0	1	1	0	5	Fair
36	Gokceli & Akkaya (2025)	Turkey	406	0	1	0	1	1	1	0	1	5	Fair
37	Gülırmak Güler et al. (2024)	Turkey	321	0	1	0	1	0	1	0	0	3	Poor
38	Hajek & Konig (2022)	Germany	3091	1	1	0	1	1	1	0	0	5	Fair
39	Hajek & König (2022)	Germany	3091	1	1	1	1	2	1	1	1	9	Good
40	Hajek & Konig (2023)	Germany	3015	1	1	0	1	1	1	1	0	6	Fair
41	Hajek & Konig (2023)	Germany	3091	1	1	0	1	0	1	1	0	5	Fair

42	Hajek & Konig (2024)	Germany	3091	1	1	0	1	1	1	1	0	6	Fair
43	Hamlaci Baskaya et al. (2024)	Turkey	978	1	1	0	1	0	1	0	1	5	Fair
44	Heeren et al. (2022)	Belgium	2080	1	1	0	1	2	1	1	1	8	Good
45	Heeren et al. (2023)	Belgium/France/Switzerland	874	1	1	0	1	0	1	0	1	5	Fair
46	Heinzel et al. (2023)	Germany	486	1	1	0	1	0	1	0	1	5	Fair
47	Henschel et al. (2025)	Germany	322	1	1	0	1	0	1	0	1	5	Fair
48	Hervè & Marsat (2023)	France	671	1	1	0	1	0	1	0	1	5	Fair
49	Hogg et al. (2021)	Australia	334	0	1	0	1	0	1	1	0	4	Poor
21	Hogg et al. (2023)	Australia	530	1	1	0	1	1	1	0	1	6	Fair

50	Hogg et al. (2024)	Australia	530	1	1	0	1	1	1	0	0	5	Fair
51	Holler et al. (2025)	—	47	1	0	0	1	0	1	1	1	5	Fair
52	Innocenti et al. (2023)	Italy	150	1	0	0	1	0	1	1	1	5	Fair
53	Innocenti et al. (2023)	Italy	394	1	1	0	1	0	1	1	1	6	Fair
54	Jalin et al. (2024)	France	522	1	1	0	1	0	1	0	1	5	Fair
55	Jalin et al. (2025)	France	262	1	0	0	1	1	1	0	1	5	Fair
56	Jang et al. (2023)	Korea	459	1	1	0	1	0	1	0	1	5	Fair
57	Jimenez-Vazquez (2025)	Spain	1065	1	1	0	1	1	1	0	1	6	Fair
58	Kabasakal-Cetin (2023)	Turkey	605	1	1	0	1	1	1	1	0	6	Fair
59	Karl & Stanley (2024)	Australia	287	1	0	0	1	0	1	1	1	5	Fair

60	Kaya et al. (2025)	Turkey	1126	0	1	0	1	0	1	0	1	4	Poor
61	Kenstler (2025)	USA	169	0	0	0	1	0	1	1	1	4	Poor
62	Kos et al. (2025)	Slovenia	324	1	1	0	1	0	1	1	1	6	Fair
63	Kratz & McEwan (2025)		151	1	0	0	1	0	1	1	1	5	Fair
64	Kryazh & Baranov (2025)	Ukraine	446	1	1	0	1	1	1	1	1	7	Fair
65	Larionow et al. (2022)	Poland	603	2	1	0	1	1	1	1	1	9	Good
66	Larionow et al. (2022)	Poland	420	2	1	0	1	1	1	1	1	9	Good
67	Larionow et al. (2022)	Poland	634	2	1	0	1	2	1	1	1	10	Good
68	López-García et al. (2025)	Spain	308	1	1	0	1	0	1	1	1	6	Fair
69	Lutz et al. (2023)	Canada	132	1	0	1	1	2	1	1	1	8	Good

70	Madune me et al. (2024)	USA	398	1	1	0	1	2	1	1	1	8	Good
71	Maral et al. (2025)	Türkiye	392	1	1	0	1	1	1	1	1	7	Good
72	Mathers-Jones & Todd (2023)	Australia	96	1	0	0	1	1	1	1	1	6	Fair
73	Memiç-İnan et al. (2025)	Türkiye	736	1	1	0	1	1	1	1	1	8	Good
74	Micoulau d-Franchi et al. (2023)	France		1	1	0	1	1	1	1	1	8	Good
75	Mohamm ed et al., 2025	Iraq (Kurdistan Region)	385	1	1	0	1	0	1	1	1	6	Fair
76	Orrù et al. (2024)	Italy	351	1	1	0	1	1	1	1	1	7	Good
77	Parmenti er et al. (2023)	France	431	1	1	0	1	1	1	1	1	7	Fair

78	Plohl et al., 2023	Slovenia	442	1	1	0	1	1	1	1	1	8	Good
79	Reyes et al. (2021)	Philippines	433	1	1	0	1	1	1	1	1	7	Fair
80	Rocchi et al. (2023)	Italy	335	1	1	0	1	2	1	1	1	8	Good
81	Rodríguez Quiroga et al. (2024)	Argentina & Spain	1,538	1	1	0	1	1	1	1	1	8	Good
82	Sampaio et al. (2023)	Portugal	623	1	1	0	1	2	1	1	1	8	Good
83	Simon et al. (2022)	Philippines	452	1	1	0	1	0	1	1	1	6	Fair
84	Skeiryte & Liobikiene (2025)	Lithuania	705	1	1	0	0	0	0	1	0	4	Poor
85	Soomro et al. (2024)	China	163	1	0	0	1	0	1	1	1	5	Fair
86	Subaşı-Turgut & Öztürk (2025)	Turkey	367	1	1	0	1	0	1	1	1	6	Fair

87	Tam et al., 2023	China, India, Japan, USA	4000	2	1	0	1	2	1	1	1	9	Good
88	Trifunović & R ajčević (2024)	Bosnia and Herzegovina	40	0	0	0	1	0	1	1	1	4	Poor
89	Tucholska et al. (2024)	Poland	333	1	1	0	1	1	1	1	1	7	Fair
90	Vecina et al. (2025)	Spain	1911	1	1	0	1	1	1	1	1	7	Fair
91	Weimann & Opaliński (2024)	Poland	431	0	1	0	1	0	1	1	0	4	Poor
92	Whitmarsh et al. (2022)	UK	1338	1	1	0	1	1	1	1	1	7	Fair
93	Wullenkord et al., 2021	Germany	1011	2	1	0	1	1	1	1	1	9	Good
94	Yeşildere Sağlam & Mızrak Şahin (2025)	Turkey	456	1	1	0	1	1	1	1	1	7	Fair
